# Identification of pyridine analogs as new predator-derived kairomones

**DOI:** 10.3389/fnins.2015.00253

**Published:** 2015-07-28

**Authors:** Julien Brechbühl, Fabian Moine, Monique Nenniger Tosato, Frank Sporkert, Marie-Christine Broillet

**Affiliations:** ^1^Department of Pharmacology and Toxicology, Faculty of Biology and Medicine, University of LausanneLausanne, Switzerland; ^2^University Center of Legal Medicine, Lausanne-GenevaLausanne, Switzerland

**Keywords:** olfaction, Grueneberg ganglion, predator scents, blood pressure, HS-SPME, GC-MS analysis, calcium imaging, behavior

## Abstract

In the wild, animals have developed survival strategies relying on their senses. The individual ability to identify threatening situations is crucial and leads to increase in the overall fitness of the species. Rodents, for example have developed in their nasal cavities specialized olfactory neurons implicated in the detection of volatile cues encoding for impending danger such as predator scents or alarm pheromones. In particular, the neurons of the Grueneberg ganglion (GG), an olfactory subsystem, are implicated in the detection of danger cues sharing a similar chemical signature, a heterocyclic sulfur- or nitrogen-containing motif. Here we used a “*from the wild to the lab*” approach to identify new molecules that are involuntarily emitted by predators and that initiate fear-related responses in the recipient animal, the putative prey. We collected urines from carnivores as sources of predator scents and first verified their impact on the blood pressure of the mice. With this approach, the urine of the mountain lion emerged as the most potent source of chemical stress. We then identified in this biological fluid, new volatile cues with characteristic GG-related fingerprints, in particular the methylated pyridine structures, 2,4-lutidine and its analogs. We finally verified their encoded danger quality and demonstrated their ability to mimic the effects of the predator urine on GG neurons, on mice blood pressure and in behavioral experiments. In summary, we were able to identify here, with the use of an integrative approach, new relevant molecules, the pyridine analogs, implicated in interspecies danger communication.

## Introduction

Predators and preys interact actively and continuously using their senses to find food or to avoid being eaten (Kavaliers and Choleris, 2001). In addition to their visual and auditory appreciation of their threatening neighborhood, preys have, for example, developed elaborated olfactory abilities to detect predator scents. Indeed by eating meat, carnivores involuntary influence their own scents that will be interpreted by the prey as the presence of an impending danger (Nolte et al., 1994; Hui, 2012). Profound behavioral alterations as well as modulation of essential physiological conditions such as the increase of stress-related hormones or the elevation of the blood pressure of the preys have been, for example, observed in the presence of these predator scents (Dielenberg and McGregor, 2001; Horii et al., 2010; Takahashi, 2014). These scents are a cocktail of chemical molecules with diverse physicochemical properties and structures, which act as interspecies communicating cues and are therefore named kairomones (Brown et al., 1970). They are present in the biological secretions of predators from their anal glands, feces, or urines (Apfelbach et al., 2005; Wyatt, 2014). They are deciphered by different olfactory neurons segregated in subsystems present in the nasal cavities of the preys (Takahashi, 2014). Large arrays of predator-derived proteins acting as non-volatile kairomones can be detected by specific neurons found in the vomeronasal organ (VNO) of the prey (Papes et al., 2010; Isogai et al., 2011). On the other hand, small volatile kairomones are mainly detected by sensory neurons found in the main olfactory epithelium (MOE) or in the Grueneberg ganglion (GG) subsystems. Kairomones may have their physiological effects in preys as single chemical cues. For example, the 2-phenylethylamine (PEA) that is found at higher level in carnivore's urine than in rodent's urine (>300 μM in lion vs. < 1 μM in rat) is specifically detected by amine-sensitive neurons in the MOE (Ferrero et al., 2011; Dewan et al., 2013; Zhang et al., 2013). The 2,4,5-trimethylthiazoline (TMT) found in red fox feces (Fendt et al., 2005) is also a robust single volatile kairomone that is detected concomitantly by the dorsal MOE (Kobayakawa et al., 2007) as well as by the GG (Brechbühl et al., 2013b) to further initiate important behavioral effects (Perez-Gomez et al., 2015).

The particularity of the GG olfactory subsystem resides in its specifically tuned chemical detection abilities. Indeed, this rostral olfactory subsystem found in rodents (Grüneberg, 1973; Tachibana et al., 1990; Fuss et al., 2005; Koos and Fraser, 2005; Fleischer et al., 2006a; Roppolo et al., 2006; Storan and Key, 2006; Fleischer and Breer, 2010; Brechbühl et al., 2014) is dedicated to the detection of volatile danger molecules from both intra and interspecies origins such as alarm pheromones (Brechbühl et al., 2008; Debiec and Sullivan, 2014) and kairomones (Brechbühl et al., 2013b) which share heterocyclic sulfur- or nitrogen-containing structures (Mamasuew et al., 2011; Brechbühl et al., 2013a,b; Hanke et al., 2013). These chemical cues initiate, in the recipient animal, stress reactions (Brechbühl et al., 2013b; Matsuo et al., 2015; Perez-Gomez et al., 2015). Molecules such as the 2-*sec*-butyl-4,5-dihydrothiazole (SBT, a recently identified mouse alarm pheromone, Brechbühl et al., 2013b), the 2,6-dimethylpyrazine [2,6-DMP, found for example in the wolf (Osada et al., 2013) and the bobcat (Mattina et al., 1991)] or the 2,3-dimethylpyridine (2,3-lutidine, Mamasuew et al., 2011; Hanke et al., 2013) share this same signature and were found to initiate GG-evoked neuronal responses.

The identification of chemical molecules that encode for innate fear reactions throughout the initiation of hard-wire neuronal circuitries is a challenging aspect of neuroscience and is fundamental for studying the interspecies communication as well as the pharmacological aspect of the neuronal olfactory pathways. The difficulty in the search of new encoding danger molecules resides principally in the screening of large repertoires of chemicals found in the wild (predator scents or alerting conspecifics) as well as to the vast possibility of detecting neurons found in the nose of the prey. Indeed, after classical chemical identification, each candidate molecule needs to be evaluated individually on the animal for its potential fear-evoked reactions before testing it on olfactory neurons, which could be time and animal-consuming (Brechbühl et al., 2013b; Kiyokawa et al., 2013; Osada et al., 2013). Moreover, encoding danger cues could have diverse chemical structures and small structural variations might also provoke distinctive sensations and neurophysiological responses in the recipient animal (Araneda et al., 2000; Nara et al., 2011; Peterlin et al., 2014). Taking advantage of the chemical structural fingerprint of known GG ligands as baits (Brechbühl et al., 2013b), we here used a “*from the wild to the lab*” approach to identify new predator-derived kairomones with the hypothesis that they will initiate GG responses and generate fear-reactions in mice (Brechbühl et al., 2008, 2013b; Debiec and Sullivan, 2014; Perez-Gomez et al., 2015). We first collected urines from different carnivores and we evaluated further by a non-invasive tail-cuff approach (measurement of blood pressure) the stress responses they generated in mice. The most potent urine, the mountain lion urine, was then analyzed by HS-SPME/GC-MS. We identified in this biological fluid, new putative kairomone candidates. We focused our attention on novel small volatile molecules, in particular the 2,4-lutidine (2,4-dimethylpyridine) and its analogs, as they shared the structural GG-fingerprint and indeed generated GG-evoked neuronal responses. We finally verified their encoded danger quality in mice using both blood pressure measurements and behavioral analysis. In summary, we used an integrative approach to identify new relevant volatile molecules implicated in predator-prey communication and identified pyridine analogs as new potent kairomones.

## Materials and methods

### Animals

C57BL/6 (*Mus musculus*; Janvier Labs) and OMP-GFP mice (Potter et al., 2001) were used from pups to adult ages. The OMP-GFP is a particular gene-targeted mouse strain that expresses the green fluorescent protein (GFP) as a histological reporter under the control of the olfactory marker protein (OMP) promoter (Mombaerts et al., 1996; Potter et al., 2001) that is specifically expressed in mature olfactory sensory neurons (Margolis, 1972). Mice were killed by cervical dislocation or by CO_2_ inhalation. The experimental procedures were in accordance with the Swiss legislation and approved by the EXPANIM commitee of the Lemanique Animal Facility Network and the veterinary authority of the Canton de Vaud (SCAV).

### Urine collection and conditioning

Urine samples were directly collected at the Servion Zoo (Servion, CH) or purchased (PredatorPee, Inc., USA). They could originate from one or more than one animal. Carnivore urines were from the mountain lion (*Puma concolor*), snow leopard (*Panthera uncia*), Siberian tiger (*Panthera tigris altaica*), bobcat (*Lynx rufus*), gray wolf (*Canis lupus*), red fox (*Vulpes vulpes*), and coyote (*Canis latrans*). Attempts of urine collection were also performed from the serval (*Leptailurus serval*), artic fox (*Alopex lagopus*), artic wolf (*Canis lupus arctos*), and ferret (*Mustela putorius furo*), but the amounts of urine collected were not sufficient for further analysis. As internal control, urine of a non-carnivore, the American bison (*Bison bison*) was used. Urine samples were rapidly filtered (0.22 μm), aliquoted in sterile tubes and kept at −80°C until used.

### Measurement of the mice blood pressure by the tail-cuff approach

The indirect and non-invasive computerized tail-cuff method (Krege et al., 1995; Fox, 2007) was used to monitor simultaneously the blood pressure of 5–6 adult male C57BL/6 mice (10.6 ± 1.9 weeks). Mice were trained for the procedure during five continuous days with the affiliated investigator (Sorge et al., 2014) and the equipment (BP-2000; Visitech) in a behavioral room (23°C, normal light cycle) to limit experimental-related stress such as balloon inflation (Zhao et al., 2011). The system was composed of tree principal parts: the platform, where mice were in experimentation, the control unit, which comprised the power supply and the air pump and a computer to generate experimental protocols and to record individual waveform signals. After calibrating the tail-cuff apparatus, mice were placed on the platform in the magnetic restrainers, their tails were inserted through the cuff/pulse sensor and immobilized with adhesive tape. The platform was heated at 37–39°C to increase the detection of the oscillation waveforms generated by the blood flow rate in the tails. Each session was composed of 10 successive measurements of the systolic and diastolic pressure after the automatic determination of the pulse rate. The system used a photoplethysmographic signal waveform analysis (Shelley and Shelley, 2001; Alian and Shelley, 2014). Briefly, a red light-emitting diode (LED) illuminated the tails of the mice and changes in light absorption due to vessel dilation were detected across time and displayed as oscillating waveform signals on the computer. Diastolic and systolic pressures were perceived by monitoring the vessel dilation during the occlusion cuffs inflation (balloon inflation). The diastolic pressure was defined as the cuff pressure necessary to observe the decrease of the waveform amplitude. The systolic blood pressure was defined as the cuff inflation pressure necessary to fall below 10% of its original stable amplitude (Krege et al., 1995). The mean pressure was calculated as the mean of the measured diastolic and systolic pressure. Measurements obtained in the presence of excessive animal movements were discarded. For each automatized session, mean individual pressures (diastolic, systolic and mean pressure) were obtained as the average of the 10 attempted measurements.

### Screening for volatile bioreactive samples by the tail-cuff approach

Measurements of blood pressure were performed to evaluate the natural stressful stimuli (Dielenberg et al., 2001) in mice. Tail-cuff recording sessions were done in the morning (9.00–11.00 a.m.) and in the afternoon (1.30–3.00 p.m.). To examine the impact of the tested volatile cues on mice blood pressure, pieces of blotting papers (1 × 1 cm) were placed in front of each tested mouse without any animal contact. 100 μl of the tested substances were deposited on them. The general evaluation procedure was performed with one conditioning measuring session (blotting papers alone) followed by three continuous control sessions (Ctrl; blotting papers with water) and three continuous sessions with the tested substances (Test; blotting papers with pure urine or 1% of synthetic cues in water, Hacquemand et al., 2013; Osada et al., 2013). Average of the three Ctrl sessions and the three test sessions were used to obtain the individual Ctrl and Test pressures. Each urine was evaluated once per animal to avoid any learning process (Kass et al., 2013).

### HS-SPME/GC-MS analysis

Headspace coupled to solid phase microextraction (HS-SPME) followed by gas chromatography coupled to mass spectrometry (GC-MS) was used to analyze volatile compounds released from the selected urine samples (Sporkert and Pragst, 2000; Ouyang and Pawliszyn, 2006; Brechbühl et al., 2013b; Osada et al., 2013). Briefly, for volatile analysis of the urines, a triplicate experiment (three different aliquots originating from the same urine sample) was performed with a polydimethylsiloxane-divinylbenzene portable fiber of 60 μm film thickness (PDMS-DVB, Supelco) inserted into the headspace of airtight 20 ml vials (# 8010, Agilent) containing 500 μl of the urine saturated with sodium chloride and placed on a heater (at 40°C) under constant magnetic agitation for 30 min. The same procedure was done with water to identify volatile contaminants present in the experimental procedure. Qualitative analyses of the extracted components present on the fibers were performed using a GC (6890 Plus, Agilent) coupled with a selective MS (HP 5973N, Agilent) with the analyzing software (MSD ChemStation, Agilent). The SPME fiber extracts were desorbed in the injector in splitless mode for 5 min at 250°C. The oven was set at an initial temperature of 40°C and ran for 20 min until 250°C. The MS was operated in the full-scan mode between 10 and 300 amu. The column used (DB-XLB, Agilent) measured 30 m length with a 0.25 mm I.D. and 0.25 μm film thickness with helium 5.0 as carrier gas. WileyN7 and NIST14 Mass Spectral libraries were used for chemical identification. Characteristic m/z values for each volatile of interest were obtained from reference spectra and used to verify the presence of the corresponding compound. For precise chemical identification, indicated by dots (•), similar procedures with pure reference standards (1:100,000), were used to confirm the presence of the identified cues. The specific ion (m/z = 107) at a retention time 8.285 min (RT_8.285_) was used, for example, to identify the 2,4-lutidine.

### Tissue preparation and calcium imaging

Noses from OMP-GFP mice (P1-7) of both sexes were dissected in ice-cold artificial cerebrospinal fluid (ACSF), containing 118 mM NaCl, 25 mM NaHCO_3_, 10 mM D-glucose, 2 mM KCl, 2 mM MgCl_2_, 1.2 mM NaH_2_PO_4_, and 2 mM CaCl_2_ (pH 7.4) saturated with oxycarbon gas [95% O_2_: 5% CO_2_; (vol/vol)] under a fluorescence-equipped dissecting microscope (M165 FC; Leica). For calcium imaging experiments, acute tissue slice preparations of the GG were performed (Brechbühl et al., 2008, 2014). Briefly, the tip of the nose was included in a block of low melting 4–5% agar. Coronal slices of 70 μm were generated on ice with a vibroslicer (VT1200S, Leica). Slices were selected under a fluorescent stereomicroscope (M165 FC, Leica) in accordance with their general morphology and their GFP expression. Fura-2 acetoxymethyl ester (AM) (5 μM; TEFLabs) was used as a ratiometric calcium dye. Slices were incubated with adjunction of pluronic acid (0.1%; Pluronic F-127, Invitrogen) for 60 min (37°C, 5% CO_2_) and were immobilized with a slice anchor in a bath chamber (RC-26, Warner Instruments). A bipolar temperature controller (SC-20/CL-100, Warner instruments) was used to maintain the bath temperature between 23 and 25°C. Visualizations were made under an inverted fluorescence microscope (Axio Observer.A1, Zeiss) with a 40x objective and a sensitive camera (Cool SNAP-HQ^2^, Photometrics). The software MetaFluor (MetaFluor, Visitron Systems) was used to monitor intracellular calcium variations and to acquire images (Brechbühl et al., 2011).

### Chemostimulation

Urine and synthetic cues (ordered from Sigma-Aldrich, Alfa Aesar, or Contech) were used as chemostimulants for calcium imaging experiments and were prepared fresh before each experiment directly diluted in ACSF with osmolarities situated between 285 and 290 Osm/L. The final dilution of the tested urine was at (1:1000) and the final concentration of synthetic cues was at 100 μM (Spehr et al., 2002; Brechbühl et al., 2013a). A short perfusion of extracellular potassium (KCl; 25 mM) was used as a cellular viability test. During the perfusion of ACSF, the spontaneous (Liu et al., 2012) intracellular calcium changes were considered as baseline activity. An increase twice larger than this baseline (corresponding to near 10% of the KCl response) was considered as a neuronal evoked-response (Brechbühl et al., 2013b).

### Behavioral analysis

An open field exploration test (Bailey and Crawley, 2009; Brechbühl et al., 2013b) was used to challenge the anxiety and stress-related behaviors of seven adult male C57BL/6 mice (10.6 ± 0.8 weeks). Briefly, 7 days before the beginning of the test, each mouse was isolated with food and water *ad libitum*. Room temperature was maintained at 23°C in a 12:12 h light/dark inverted cycle. Animals were trained to handling, familiar with the test arena context (a closed Plexiglas box of 45 × 25 × 19 cm) and to the presence of a piece of blotting paper (3 × 3 cm) to minimize environmental-related stress. Behavioral experiments were performed by the affiliated investigator (Sorge et al., 2014) during the nocturnal phase. They were recorded for at least 5 min from the top of the arena covered by a Plexiglas plate by an IR-sensitive HD camera under nightshot vision (infra red illumination) and subsequently analyzed offline in simple blind conditions with a video tracking system (ANY-maze, Stoelting). To assess the stress-related behaviors displayed by mice in the presence of 1% synthetic predator-derived kairomones, neutral cue (Water) or pure predator urines (200 μl on blotting paper), the number of visits in the central zone of the arena (corresponding to the half of the total arena surface Bailey and Crawley, 2009), the total walking distance, the defecation (number of fecal pellets), the number of risk assessment episodes and the total freezing duration were quantified (Vernet-Maury et al., 1984; Blanchard et al., 1990; Fendt et al., 2005; Staples et al., 2008; Brechbühl et al., 2013b; Hacquemand et al., 2013). Each tested substance sessions were compared to a control session (Ctrl) performed before with only blotting paper and used to calculate the index of each evaluated behaviors (as a percent for the central zone, the walking distance and the freezing or as a score for the defecation and the risk assessment). The automatic detection of the center of the animal was used as reference point by the video tracking system. The freezing on/off (30/40) thresholds with a minimum freezing period threshold of 500 ms were used. To minimize animal habituation, only one test session and its Ctrl session were performed per day and each tested substance was used only once per mouse (Kass et al., 2013).

### Statistics

The open source statistical package R version 3.1.2 was used. Normality and homogeneity were evaluated by the Shapiro test. Accordingly, comparisons between Ctrl and tested sessions were performed with the one-tailed paired Student's *t*-test or Wilcoxon *w*-test. Values are expressed as mean ± SEM. Significance levels are indicated as follows: ^*^p < 0.05; ^**^p < 0.01; ^***^p < 0.001; ns for non-significant.

## Results

### Measurements of the mice blood pressure revealed bioreactivity of urine samples

**Figure 1:**
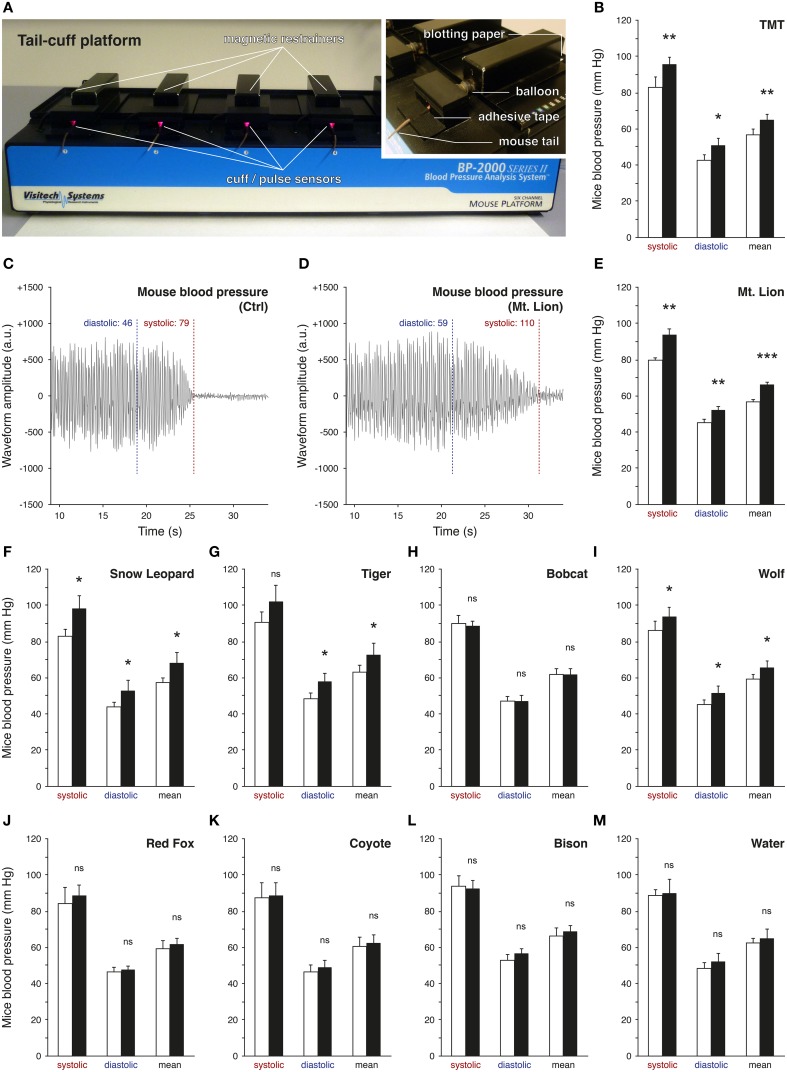
**Screening of predator urines via tail-cuff analysis**. The blood pressure of mice is analyzed by tail-cuff measurements. **(A)** Mice are placed on the tail-cuff platform, which is connected to a control unit and a computer. They are maintained in magnetic restrainers and their tails are placed in the cuff/pulse optical sensor. Details of the experimental procedure are shown; the blotting paper (tested cues), the inflatable balloon (tail pressure control) and the fixation of the tail with adhesive tape. **(B)** The system is tested here with 1% TMT (synthetic red fox kairomone), which significantly increases the mice blood pressure. **(C,D)** Examples of one measurement for the diastolic and the systolic pressures performed on the same mouse under control conditions (**C**, Ctrl) or test conditions, here exposed to the pure urine of the mountain lion (**D**, Mt. Lion). Oscillating waveforms are obtained according to the automatic analysis of the photoplethysmographic signal detected by the pulse sensors [red LED in **(A)**]. Waveform amplitudes are indicated by arbitrary units (a.u.). The mean pressure is calculated as the mean between the diastolic and the systolic pressures. **(E–M)** Tests of different carnivore urines (**E–H**, *Felidae*; **I–K**, *Canidae*), non-carnivore urine (**L**, Bison) or only water (**M**, Water). Exposure to the urine of the Mt. Lion induced the most significant increase in mice blood pressure **(E)**. Control conditions (white bars) and test sessions (black bars) are shown **(B–M)**. Five adult male mice were used **(B,E–M)**. Values are expressed as mean ± SEM; one-tailed paired *t*-test or *w*-test, ^*^P < 0.05; ^**^P < 0.01; ^***^P < 0.001; ns, not significant.

Predator urines act as natural stressors in rodents due to their kairomone content (Apfelbach et al., 2005; Fendt et al., 2005; Kobayakawa et al., 2007; Ferrero et al., 2011). Indeed, after olfactory detection, typical alterations of behaviors or increases of stress-related hormones and blood pressure have been previously observed in the presence of predator urines (Takahashi, 2014). However, significant differences between urine samples were reported depending, for example, on the period of collection (Osada et al., 2013), the origin of the species (emitter or recipient; Apfelbach et al., 2005) or the preceding diet of the predator itself (Nolte et al., 1994). Based on these reports, we took advantage of a rapid and non-invasive experimental strategy to screen bioreactive predator urines (Figure 1) by measuring the elevation of mice blood pressure with the tail-cuff approach (Figure 1A) (Krege et al., 1995). We first challenged this method by measuring the variation of the blood pressure of mice exposed to 1% TMT, a potent red fox-derived kairomone known to elicit fear in rodents (Fendt et al., 2005; Horii et al., 2010, 2013; Hacquemand et al., 2013; Takahashi, 2014). As expected significant increases in blood pressure were observed in mice between the TMT exposure and its control level (Figure 1B). We thus next measured the mice blood pressure in the presence of the volatile fraction of the different predator urines. Systolic, diastolic, and mean blood pressures were systematically measured according to the signal waveform displayed by the tail-cuff approach (Figures 1C,D) while mice were exposed to the collected carnivore urines from *Felidae* such as the mountain lion (Mt. Lion; *Puma concolor*), snow leopard (Snow Leopard; *Panthera uncia*), Siberian tiger (Tiger; *Panthera tigris altaica*) and the bobcat (Bobcat; *Lynx rufus*) (Figures 1E–H) as well as urine from *Canidae* such as the gray wolf (Wolf; *Canis lupus*), red fox (Red Fox; *Vulpes vulpes*) and coyote (Coyote; *Canis latrans*) (Figures 1I–K) used here as mice predators. We observed and confirmed by this approach that the tested predator urines were not equally efficient to induce an elevation in mice blood pressure, an early physiological parameter (Dielenberg et al., 2001). Furthermore, and consistent with previous reports, urine samples originating from the same predator species (snow leopard in our case) but collected at different moments were indeed not equivalent (data not shown; Osada et al., 2013). Nevertheless, the mountain lion emerged (Figure 1E) as the most potent source of volatile kairomones across the tested carnivore urines as its presence significantly increased mice blood pressures nearly by 15% compared to the control level for the systolic (Ctrl_systolic_: 79.6 ± 1.4 mm Hg; Mt. Lion_systolic_: 93.7 ± 3.4 mm Hg; *t*-test: ^**^), diastolic (Ctrl_diastolic_: 45.1 ± 1.7 mm Hg; Mt. Lion_diastolic_: 52.4 ± 1.7 mm Hg; *t*-test: ^**^) and mean blood pressure (Ctrl_mean_: 56.7 ± 1.4 mm Hg; Mt. Lion_mean_: 66.4 ± 1.2 mm Hg; *t*-test: ^***^). As non-carnivore sample and internal procedure controls (Fendt, 2006), urine from a *Bovidae* (Bison; *Bison bison*) (Figure 1L) as well as water (Figure 1M) were respectively used and were indeed not able to increase blood pressure in mice. The tail-cuff method, in addition to the blood pressure measurements, also allowed the evaluation of the animal heart rates (HR) by using the frequencies of the waveform signals (Figures 1C,D). Interestingly and consistent with previous observations (Dielenberg et al., 2001), we saw here for the TMT and for the fear-inducing predator urines (Mt. Lion, Snow Leopard, Tiger and Wolf), a systematic negative drift of the HR (−8.8±6.3%) between the control and the tested situations. This physiological criterion was not affected by predator urines that did not induce an elevation of blood pressure nor by water (+1.6 ± 0.9%). With this first technical approach, we thus screened bioreactive urines and selected the one issued from the mountain lion as the most potent source of volatile kairomones for further investigations.

### Identification of putative volatile kairomones by HS-SPME/GC-MS

**Figure 2:**
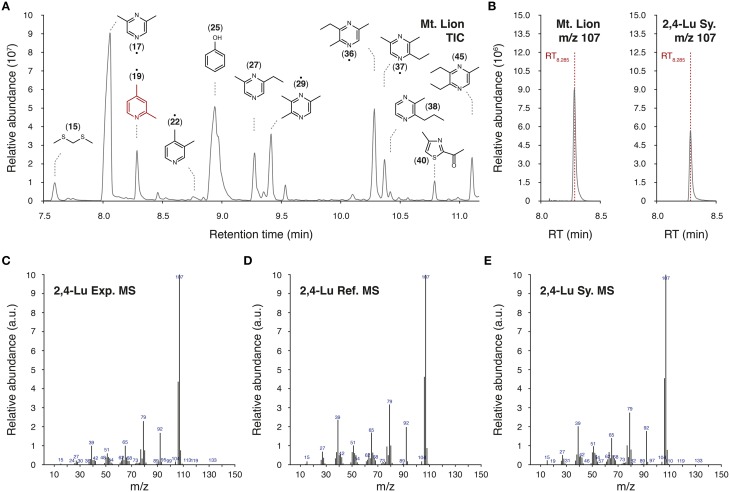
**Identification of pyridine analogs as putative kairomones**. **(A)** Representative part of a total ion chromatogram (TIC) of volatiles emitted from the urine of the mountain lion obtained by HS-SPME/GC-MS. Chemical formulas of a selection of volatiles are indicated above peaks with their affiliated ID number. When not mentioned as precise chemical identification (•), molecules have been tentatively identified by WileyN7/NIST14 mass spectral libraries. The 2,4-lutidine (2,4-Lu; ID19) is highlighted in red. **(B–E)** Detailed GC-MS analysis of the HS-SPME fiber extract. Here, the identification of the 2,4-lutidine is shown as an example. The mass charge ratio (m/z) value of 107 was used for the precise identification of the 2,4-lutidine (2,4-Lu) thanks to its retention time of 8.285 min (RT_8.285_) in the urine of the mountain lion (**B**, Mt. Lion) and for the synthetic 2,4-lutidine (**B**, Sy.). Experimental m/z spectrum (**C**, Exp. MS) is compared with the reference mass spectrum (**D**, Ref. MS) found in the WileyN7/NIST14 mass spectral libraries as well as with the corresponding mass spectrum of the synthetic cue (**E**, Sy. MS).

We used a HS-SPME/GC-MS approach to extract and analyze volatile chemicals present in the urine of the mountain lion (Table 1 and Figure 2). In our experimental conditions, we identified 98 volatile candidates, 61 of them were referenced in the chemical databases. Among them, numerous pyrazine and thiazolic analog structures were detected (Figure 2A). A couple of compounds were previously found in predator urines and/or shown to act as kairomones in mice (Zhang et al., 2005; Brechbühl et al., 2013b; Osada et al., 2013). To identify new putative kairomones, we hypothesized that potential new GG ligands would encode for innate fear-reactions (Brechbühl et al., 2008, 2013b; Debiec and Sullivan, 2014; Perez-Gomez et al., 2015). We thus focused on volatile candidates that also share the heterocyclic sulfur- and/or nitrogen-containing structure related to the detection ability of this olfactory subsystem (Mamasuew et al., 2011; Brechbühl et al., 2013b). We were particularly interested by the pyridine analogs as they share this chemical signature and they were, for another derivative, previously found to evoke neuronal responses in the GG (Mamasuew et al., 2011; Hanke et al., 2013). Interestingly, the 2,4-lutidine (2,4-Lu; also known as 2,4-dimethylpyridine) identified according to its retention time (RT_8.285_; Figures 2A,B) and its corresponding mass spectra (MS; Figures 2C–E) had a relative abundance of ionized product similar to the one observed for the pyrazine structures (Figure 2A). We evaluated its concentration in the original urine of the mountain lion to be in the range of 60–120 μM by comparison with the HS-SPME/GC-MS detection of its synthetic cue (2,4-Lu Sy.; Figure 2B). This value corresponds to concentrations previously observed for other kairomones in predator urines (Ferrero et al., 2011). Thus, from the molecular structures detected in the urine of the mountain lion and the chemical identification process we selected pyridine analogs as new putative predator-derived kairomones.

**Table 1:** **HS-SPME/GC-MS analysis of the urine of the mountain lion**.

**RT**	**ID**	**Molecule**	**Usual name**
2.141	1	1-Propanol	Propyl alcohol
4.254	2	3-Buten-1-ol, 3-methyl-	Isoprenol
4.678	3	1,2-Propanediol	Propylene glycol
4.775	4	1H-Pyrrole	–
5.314	5	2-Buten-1-ol, 3-methyl-	Prenol
5.870	–	Unidentified compound	–
6.304	6^•^	Pyridine, 4-methyl-	4-Picoline
6.450	7	Pyrazine, 2-methyl-	–
6.617	8	2-Pentenal, 2-methyl-	–
6.728	9	Ethanol, 2-(methylthio)-	–
6.989	10	Cyclopentanone, 3-methyl-	–
7.069	11	Thiazole	–
7.149	12	1-Hexanol	Hexyl alcohol
7.409	–	Unidentified compound	–
7.489	13^•^	Pyridine, 2,6-dimethyl-	2,6-Lutidine
7.531	14	3-Heptanone	Butyl ethyl ketone
7.590	15	Methane, bis(methylthio)-	2,4-Dithiapentane
7.743	16	Oxime-, methoxy-phenyl-	–
8.084	17^•^	Pyrazine, 2,6-dimethyl-	–
8.195	18	1H-Pyrrole, 2,5-dimethyl-	–
8.285	19^•^	Pyridine, 2,4-dimethyl-	2,4-Lutidine
8.525	20	1-Butanamine, N-butyl-	Dibutylamine
8.674	21	Pyridine, 2-ethyl-6-methyl	2-Picoline, 6-ethyl-
8.754	22^•^	Pyridine, 3,4-dimethyl-	3,4-Lutidine
8.845	23	Cyclopentanone, 3-ethyl-	–
9.144	24	2-Octanone	–
8.942	25	Phenol	–
9.171	26	1H-Pyrrole, 2,3,5-trimethyl-	–
9.266	27	Pyrazine, 2-ethyl-6-methyl-	–
9.338	28	Pyrazine, 2-ethyl-5-methyl-	–
9.415	29^•^	Pyrazine, 2,3,5-trimethyl -	–
9.627	–	Unidentified compound	–
9.651	30	Thiazole, 2-acetyl-	
9.707	31	Ethane, 1,2-bis(methylthio)-	2,5-Dithiahexane
9.821	32	Pyrazine, 2-isopropyl-5-methyl-	–
9.856	33	Furan, 2-Acetyl-5-methyl-	–
10.009	34	Phenol, 2-methyl-	*o*-Cresol
10.100	–	Unidentified compound	–
10.190	–	Unidentified compound	–
10.273	35	Phenol, 4-methyl-	*p*-Cresol
10.284	36^•^	Pyrazine, 3-ethyl-2,5-dimethyl-	–
10.371	37^•^	Pyrazine, 2-ethyl-3,5-dimethyl-	–
10.419	38	Pyrazine, 2-methyl-3-propyl-	–
10.489	39	Pyrazine, 2-methyl-5-propyl-	–
10.694	–	Unidentified compound	–
10.795	40	Thiazole, 2-acetyl-4-methyl-	–
10.909	41	Pyrazine, 2-methyl-6-propyl-	–
10.965	42	2-Propanone, 1-phenyl-	Phenylacetone
10.986	43	1H-Pyrrole-2-carboxaldehyde, 3,4-dimethyl-	–
11.014	44	2-Cyclohexen-1-one, 3,5,5-trimethyl-	Isophorone
11.104	45	Pyrazine, 2,3-diethyl-5-methyl-	–
11.239	46	Pyrazine, 2,5-dimethyl-3-propyl-	–
11.333	47	Pyrazine, 3,5-dimethyl-2-propyl-	–
11.451	–	Unidentified compound	–
11.507	–	Unidentified compound	–
11.538	48	Pyrazine, 2-acetyl-3,5-dimethyl-	–
11.834	–	Unidentified compound	–
11.875	49	Pyrazine, 2,5-diethyl-3,6-dimethyl-	–
11.921	–	Unidentified compound	–
11.976	–	Unidentified compound	–
12.022	50	Pyrazine, 2,3,5-trimethyl-6-propyl-	–
12.077	51	Pyrazine, 2,5-dimethyl-3-(1-propenyl)-, (Z)-	–
12.119	-	Unidentified compound	–
12.136	52	Benzothiazole	–
12.160	–	Unidentified compound	–
12.220	53	Pyrazine, 2-Isopropenyl-3,6-dimethyl-	–
12.296	–	Unidentified compound	–
12.345	–	Unidentified compound	–
12.522	54	Pyrazine, 2-methyl-3-propyl-	–
12.657	–	Unidentified compound	–
12.800	55	1H-Indole	–
13.015	56	3,3-dimethyl-4,5-dithiahexan-1-ol	–
13.081	–	Unidentified compound	–
13.148	–	Unidentified compound	–
13.353	57	Pyrazine, 3,6-dipropyl-2,5-dimethyl-	–
13.429	58	Pyrazine, 3-isopentyl-2,5-dimethyl-	–
13.592	59	Pyrazine, 2,6-dimethyl-3-(2-methyl-1-butyl)-	–
13.634	–	Unidentified compound	–
13.707	–	Unidentified compound	–
13.822	60	1H-Benzimidazole, 2,5-dimethyl-	–
13.864	–	Unidentified compound	–
13.923	–	Unidentified compound	–
13.978	61	5,9-Undecadien-2-one, 6,10-dimethyl-, (E)-	Geranyl acetone
14.142	–	Unidentified compound	–
14.194	–	Unidentified compound	–
14.246	–	Unidentified compound	–
14.284	–	Unidentified compound	–
14.406	–	Unidentified compound	–
14.548	–	Unidentified compound	–
14.736	–	Unidentified compound	–
14.931	–	Unidentified compound	–
15.031	–	Unidentified compound	–
15.424	–	Unidentified compound	–
15.978	–	Unidentified compound	–
16.046	–	Unidentified compound	–
16.070	–	Unidentified compound	–
16.547	–	Unidentified compound	–
18.907	–	Unidentified compound	–

Volatiles from the urine of the mountain lion are listed according to their retention time (RT) expressed in minutes. Sixty-one referenced volatiles were detected in our experimental conditions. The identification number (ID), the chemical nomenclature (Molecule) and the usual name, if existing, of the identified molecules are mentioned. Detected compounds with no attributed name found in the Wiley7N or the NIST14 libraries are listed as “Unidentified compound.” Precise chemical identifications have been performed for the volatiles marked with dots

(•) *, otherwise they have been tentatively identified from the libraries*.

### Pyridine analogs mimicked the systemic effects of predator urine in mice

**Figure 3:**
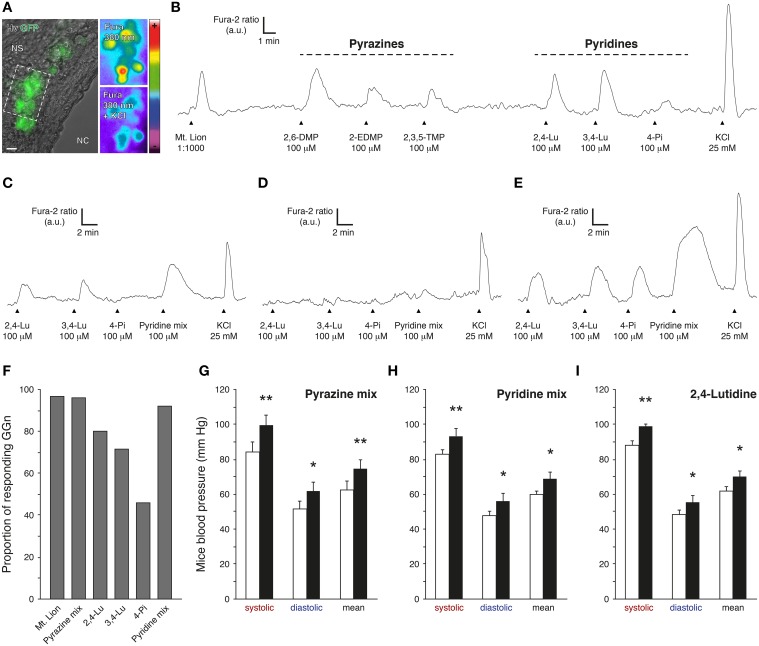
**Pyridine analogs are sufficient to mimic the systemic effects of predator urine in mice**. **(A)** Coronal slice from the Grueneberg ganglion (GG) of an OMP-GFP mouse loaded with Fura-2AM. GG neurons (GGn) are localized between the nasal cavity (NC) and the nasal septum (NS). According to their intrinsic GFP fluorescence, GGn can be selected and observed either under Hoffman modulation view (Hv) or 380 nm in color-encoded map for unbound Fura. Here, a typical intracellular calcium increase induced by a control pulse of KCl (25 mM) observed before and at the peak in selected GGn (dashed rectangle). Scale bar, 10 μm in **(A)**. **(B)** Representative continuous recording of a GGn responding to diluted urine of the mountain lion (Mt. Lion; 1:1000), to a set of pyrazines (2,6-dimethylpyrazine, 2,6-DMP; 2-ethyl-3,5-dimethylpyrazine, 2-EDMP; 2,3,5-trimethylpyrazine, 2,3,5-TMP; 100 μM) and to a set of pyridines (2,4-lutidine, 2,4-Lu; 3,4-lutidine, 3,4-Lu; 4-picoline, 4-Pi; 100 μM). **(C–E)** Examples of GGn with differential pattern of pyridine-evoked responses. **(F)** Proportion of responding GGn to the different tested cues. The Pyrazine mix (2,6-DMP, 2-EDMP, 2,3,5-TMP; 100 μM) and the Pyridine mix (2,4-Lu, 3,4-Lu, 4-Pi; 100 μM) were both able to initiate similar numbers of GGn responses comparable to the one observed with the diluted urine of the mountain lion (1:1000). A total of 60 viable GGn isolated from 6 mice (P1-7) were used **(A–F)**. Fluorescence intensity Fura-2 ratio = F340/F380 is indicated by arbitrary units (a.u.); times are indicated by horizontal bars in **(B–E)**. **(G–I)** Mice blood pressure analyzes by tail-cuff measurements for the Pyrazine mix **(G)**, the Pyridine mix **(H)** or the 2,4-lutidine **(I)** at a dilution of 1%. Control conditions (white bars) and test sessions (black bars) are shown **(G–I)**. Six adult male C57BL/6 mice were used **(G–I)**. Values are expressed as mean ± SEM; one-tailed paired *t*-test or *w*-test, ^*^P < 0.05; ^**^P < 0.01; ns, not significant.

The 2,4-lutidine and two other pyridine analogs, the 3,4-lutidine (3,4-Lu; also known as 3,4-dimethylpyridine) and the 4-picoline (4-Pi; also known as 4-methylpyridine) (Table 1) were selected for further evaluation of their potential fear-like reactivity in mice (Figure 3). In a first set of experiments, we performed calcium imaging on GFP-expressing GG neurons from mouse coronal slices previously incubated in Fura-2AM, a ratiometric calcium sensitive dye (Brechbühl et al., 2008, 2013b) (Figure 3A). GG slices were continuously perfused with oxycarbonated artificial cerebrospinal fluid (ACSF) at room temperature. Chemical stimulations were performed with a perfusion system and by direct dilution of the tested cues in ACSF. Fura-loaded GG neurons were identified by their intrinsic green fluorescence and by their specific morphology (Brechbühl et al., 2014). GG-evoked responses were observed and graphed by the variation of the Fura-2 ratio fluorescence (Fura-2 ratio) and brief stimulation of KCl was used to evaluate the GG neuronal viability (Figures 3B–E). In a total of six individual experiments (*n* total of recorded and viable GG neurons = 60), we found that successive stimulations with the selected pyridines initiated reproducible and reversible responses in GG neurons at a concentration of 100 μM (Brechbühl et al., 2013b), a concentration similar to the one previously estimated in the tested urine of the mountain lion. As a control, we found that the pyridine-sensitive GG neurons were also able to respond to the biological source of these pyridines, the urine of the mountain lion (Mt. Lion, 1:1000) as well as to pyrazines with a chemical structure related to the ones detected by the GG such as the 2,6-dimethylpyrazine (2,6-DMP; Mamasuew et al., 2011; Brechbühl et al., 2013b; Osada et al., 2013), the 2-ethyl-3,5-dimethylpyrazine (2-EDMP) or the 2,3,5-trimethylpyrazine (2,3,5-TMP; Mamasuew et al., 2011; Osada et al., 2013) (Figure 3B). Interestingly, the tested pyridines were not equally efficient in evoking neuronal responses in GG neurons (Figures 3C–F). For example, we recorded GG neurons responding partially (Figure 3C), not responding (Figure 3D) or fully responding (Figure 3E) to the three different pyridines. Among all the observations, the 2,4-lutidine emerged as the most potent single pyridine in terms of the number of GG-evoked responses (*n* = 28∕35; Figure 3F). The perfusion of a mix of the three pyridines (Pyridine mix; *n* = 24∕26) was equally efficient to a mix composed by the three tested pyrazines (Pyrazine mix; *n* = 23∕24) or to the urine of the mountain lion (*n* = 58∕60; Figure 3F). In summary, this first experimental approach showed that pyridine analogs act as GG stimuli.

The Grueneberg ganglion subsystem is implicated in olfactory danger communication (Brechbühl et al., 2008, 2013b; Debiec and Sullivan, 2014; Perez-Gomez et al., 2015). These pyridine-evoked GG responses should therefore induce fear-like reactions in the recipient animal. We thus challenged mice with these pyridine analogs. Mice blood pressures were measured in the presence of 1% pyrazine mix (Figure 3G) or 1% pyridine mix (Figure 3H). We observed, with this approach, that pyrazine analogs as well as pyridine analogs were sufficient to mimic the fear-like reaction observed with the pure source of predator kairomones, the urine of the mountain lion (Figure 1E). Furthermore, at the single chemical level, the 2,4-lutidine alone (1% 2,4-lutidine) emerged as potent fear-inducer as it was sufficient to significantly increase mice blood pressure (Figure 3I). Interestingly, we also saw with the tail-cuff approach that these synthetic cues generated a similar systematic negative drift of the HR (−9.7±2.0%) as the one observed for the mountain lion urine (−5.5±2.0%). Thus, we showed here that pyridine analogs not only activated GG neurons at physiological concentrations but that they were able and sufficient to mimic the systemic responses observed with the predator urine.

### Pyridine analogs evoked innate fear reactions in mice

**Figure 4:**
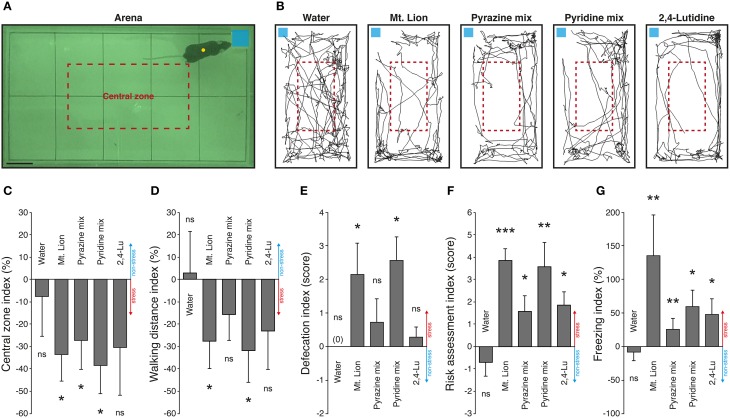
**Pyridine analogs generate stress-related behaviors in mice**. An open field exploration test is used to evaluate the innate fear reactions of mice. **(A)** The behavioral arena observed during nocturnal phase. The limits of the arena zone (black rectangle) and the central zone (red dashed rectangle) are shown. Mice are automatically tracked (yellow circle) in the presence of different tested cues deposited on a blotting paper (blue square). Scale bar, 5 cm in **(A)**. **(B)** Representative tracking distances covered by the same mouse in the presence of a neutral cue (Water), pure mountain lion urine (Mt. Lion), a mix of pyrazines (Pyrazine mix; 2,6-DMP, 2-EDMP, 2,3,5-TMP; 1%), a mix of pyridines (Pyridine mix; 2,4-Lu, 3,4-Lu, 4-Pi; 1%) or 2,4-lutidine (2,4-Lu; 1%). **(C–G)** Stress-related behaviors are quantified according to the control session and displayed as indexes. Stress (red) or non-stress (blue) tendencies are displayed on graphics. The number of visits in the central zone of the arena **(C)**, the total walking distance recorded during 5 min **(D)**, the defecation (number of fecal pellets) **(E)**, the risk assessment episodes **(F)** and the freezing behaviors **(G)** were quantified. Innate fear reactions were observed for the mountain lion urine, the mixes of pyrazines and pyridines and for the 2,4-lutidine. Seven adult male mice were used **(C–G)**. Values are expressed as mean ± SEM; one-tailed paired *t*-test or *w*-test, ^*^P < 0.05; ^**^P < 0.01; ^***^P < 0.001; ns, not significant.

Physiological alterations such as blood pressure increases and GG neuronal activations are consistent evidences of fear-like encoding cues, but to verify the ability of pyridine analogs to evoke innate fear in mice, we challenged them in an open field behavioral test (Figure 4). Mice were placed in an arena in the presence of the different tested substances deposited on a blotting paper (Figure 4A). They were observed in the presence of a neutral cue (Water) (Dewan et al., 2013; Perez-Gomez et al., 2015) or in the presence of mountain lion urine (Mt. Lion) (Dewan et al., 2013) or 1% pyrazine mix (Pyrazine mix) (Osada et al., 2013) used as internal non-aversive and stress-inducing references to evaluate the potency of the candidate kairomones 1% pyridine mix (Pyridine mix) and 1% 2,4-lutidine (2,4-Lu) to generate fear. We first tracked the movements of the mice during sessions of 5 min under control and test situations (Figure 4B). We observed a decrease of the exploratory behavior performed in the central zone arena (Figure 4C), as well as a decrease of the general walking activity previously described as anxiety traits (Vernet-Maury et al., 1984; Bailey and Crawley, 2009; Brechbühl et al., 2013b) (Figure 4D). Additional important stress-related criteria such as increases of defecation (Figure 4E), risk assessment episodes (Figure 4F) and freezing behaviors (Figure 4G) were also displayed (Blanchard et al., 1990; Apfelbach et al., 2005; Fendt et al., 2005; Brechbühl et al., 2008; Staples et al., 2008; Hacquemand et al., 2013). In summary, the neutral cue used here was not able to generate any stress-related reactions in mice. On the other hand, strong stress responses were observed for the mountain lion urine and, for both mixes of synthetic cues, the pyrazines, and the pyridines. Consistent with our previous observations done on GG slices or with blood pressure measurements, the 2,4-lutidine alone was also able to generate stress reactions in mice especially for the risk assessment and for the freezing behaviors (Figures 4F,G). Thus, as for pyrazine analogs (Osada et al., 2013), pyridine analogs could therefore be considered as new predator-derived kairomones as they initiate innate fear reactions in mice.

## Discussion

Odorants present in the environment provide relevant information about the context in which the animals live. Preys, for example, detect the presence of their predators and avoid fatal encounters through the deciphering of specific predator scents (Isogai et al., 2011). This olfactory ability is crucial for their survival and is mediated by parallel and/or complementary olfactory neuronal pathways (Ma, 2010; Takahashi, 2014). After screening the effectiveness of fear-inducing predator urines, we focused our attention on volatiles that could be detected by the Grueneberg ganglion based on their structural fingerprint (Brechbühl et al., 2013b). We found pyridine analogs that shared this feature as new putative predator-derived kairomones and showed that these candidate molecules were indeed detectable by mice GG neurons. Nevertheless, they could also activate additional neuronal pathways as they have, for humans, a strong and aversive smell (Brechbühl et al., 2013b). Olfactory neurons in the VNO (Osada et al., 2013) or in the MOE (Matsuo et al., 2015) could therefore be implicated alongside with the GG neurons in the deciphering of these molecules and allow, for example, fine behavioral adjustment in the recipient animal (Perez-Gomez et al., 2015). The use of mouse models with targeted invalidation of olfactory subsystems could clarify their specific involvement in the pyridine analog recognition. The analysis of stress-evoked effects recorded in the presence of pyridine analogs in mice with a genetic deletion of the essential MOE component, the cyclic nucleotide-gated channel A2 (CNGA2) (Brunet et al., 1996), or the key VNO-signaling element, the transient receptor potential channel type 2 (TRPC2) (Stowers et al., 2002; Papes et al., 2010) as well as in mice with surgical ablation of the GG (Brechbühl et al., 2008) would be of particular interest. Nevertheless, we found that pyridine analogs were able and sufficient to initiate blood pressure increase, a known early physiological response to predator scents (Dielenberg et al., 2001) and to evoke fear-reactions in the recipient mice. Pyridine analogs could therefore be considered as new chemical actors in the predator-prey communication.

The identification of single kairomones encoding for innate fear reactions in prey is a fundamental ethological challenge. For our HS-SPME/GC-MS experiments, we chose a PDMS-DVB fiber according to its physical characteristics and ability to trap numerous volatiles of interest (Soso et al., 2014). Supplementary analyses performed either with other SPME fibers or alternative chemical detection systems would probably allow identification of additional putative kairomones. Indeed, not all molecules can be adsorbed by our method of identification, consistent here with our inability to detect known kairomones present in the urine of the mountain lion such as the PEA (Ferrero et al., 2011). Yet based on the chemical structures of the detected molecules (Hui, 2012), the present study proposes multiple GG-activating kairomone candidates such as numerous pyrazine or pyrrole analogs. These compounds as well as the ones not referenced in the used chemical libraries deserve additional investigations. Confirmation of the presence of pyridine analogs in other predator urines and their encoding danger quality for other rodentia preys would also reveal the ubiquity of these molecules in the predator-prey communication context (Brechbühl et al., 2014).

Predator scents are complex cocktails of molecules with different physicochemical properties. Here we first used the non-invasive tail-cuff approach to demonstrate the physiological effects of fear-like responses in mice exposed to volatile sources. We used this approach to screen fear-inducing urines and we identified the urine of the mountain lion as the most potent source of volatile kairomones as it generated in mice the most significant increase in blood pressure. These observations were also correlated with the GG neuronal responses evoked by the different predator urines in calcium imaging experiments (data not shown). Nevertheless, predator urines that did not induce fear reactions in mice, as observed by this technical approach, could also be the source of non-volatile kairomones (Papes et al., 2010; Isogai et al., 2011) and could induce strong aversive effects in classical behavioral experiments in mice. Compared to other non-invasive analysis of fear reactions such as the behavioral analysis (e.g., freezing, risk assessment) (Kobayakawa et al., 2007; Brechbühl et al., 2013b), the tail-cuff method allows the collection of rapid and still robust results, and thus is an interesting alternative strategy for screening large arrays of fear-inducing cues such as predator urines or kairomone candidates. Nevertheless, pleasant and unpleasant odorants such as lavender oil or the non-predatory odor butyric acid could induce decreases or only extremely weak increases of blood pressure respectively (Major and Silver, 1999; Tanida et al., 2006; Horii et al., 2010). Additional methods could therefore be used to confirm the innate fear-inducing effects of the candidate cues screened by the tail-cuff method. Here we chose the behavioral analysis, but alternative methods could also be performed such as telemetry measurements (Dielenberg et al., 2004), that could provide additional and long-lasting physiological values (e.g., general animal activity, animal temperature) and/or invasive approaches such as the analysis of the animal corticosterone level (Thomas et al., 2006; Ferrero et al., 2011; Brechbühl et al., 2013b).

In addition to the blood pressure measurements, the tail-cuff method also allows the evaluation of the animal heart rates. This physiological value was reported to be less relevant for fear-induced situations (Dielenberg and McGregor, 2001; Takahashi, 2014) and thus was not primarily used in this study as a criterion for screening and evaluating fear-inducing urines. Interestingly and consistently with previous observations (Dielenberg et al., 2001), we saw, as a whole, for the fear-inducing predator urines and the synthetic cues, a systematic drift of the HR (−9.1±3.8%) between the control and the tested situations (Ctrl_meanHR_: 458.4 ± 33.4 bpm; Test_meanHR_: 411.7 ± 27.1 bpm; *t*-test: ^*^). This physiological trait deserves further investigations and could be, for example, a contextual adaptation of the animal to hiding strategies (Kavaliers and Choleris, 2001; Barber and Conner, 2007; Pereira et al., 2012).

Predator-derived kairomones such as the pyrazine (Osada et al., 2013) or the pyridine analogs appear to be important contributors to the predator-prey chemical communication. For the prey, the Grueneberg ganglion acts as a specialized olfactory subsystem implicated in the detection of these meat-derived cues (Brechbühl et al., 2013a,b). The biochemical pathways of these kairomones are taking place in the predator guts. They are unknown but mostly depend of the meat-metabolism produced by the digestion and the species-dependent commensal microflora (Brown, 1979; Schellinck and Brown, 2000; Apfelbach et al., 2005). Their chemical formations could use different amino acids as origins to generate convergent structures (Suyama and Adachi, 1980; Yu and Zhang, 2010; Hui, 2012). We may here speculate that danger cues derivating from similar biochemical pathways would have analogous chemical structures and thus could be deciphered by related GG neuronal pathways (Brechbühl et al., 2013a). Here we show that pyridine and pyrazine analogs that are chemically similar (nitrogen containing molecules) were detected by the same GG neurons. They could derive from conserved biochemical pathways. On the other hand, danger molecules that also evoked GG responses and innate fear reactions such as the mouse alarm pheromone SBT, the red fox kairomone TMT (nitrogen and sulfur containing molecules) or the stoat kairomone 2-PT (2-propylthietane; sulfur containing molecule) have distinctive chemical structures and could therefore use alternative GG signaling (Brechbühl et al., 2008, 2013a,b; Perez-Gomez et al., 2015). Indeed, more than one receptor or signaling pathway are expressed in GG neurons (Fleischer et al., 2006b, 2007, 2009; Liu et al., 2009, 2012; Brechbühl et al., 2013a, 2014) and could therefore be used to detect the large array of danger cues in a single GG neuron differently from the single recognition cascades that take place in the neurons of the MOE or VNO (Touhara and Vosshall, 2009; Ma, 2012). Consistent with previous reports (Mamasuew et al., 2011; Hanke et al., 2013), this characteristic is reinforced in the present study by the differential patterns of pyridine-evoked responses observed in GG neurons. Calcium imaging experiments revealed the 2,4-lutidine as the most potent tested pyridine analog. Nevertheless, using a mix of pyridine analogs as a source of GG stimulus, we found that both the proportion of GG-evoked responses as well as the cellular intensity of the response itself were increased. These cumulative neuronal effects are consistent with the potential expression of multiple signaling pathways in a single neuron (Yu and Zhang, 2014; Yu et al., 2014) and they could be an evolutionary conserved feature to increase the coding complexity and broadening of the neuronal tuning profile (Spehr and Leinders-Zufall, 2005; Brechbühl et al., 2013a).

Our study gives new insights into how organisms interact and communicate chemically. Indeed, physiological impacts could be observed in the prey after the olfactory detection of chemical danger cues occurred. This prey's ability to detect kairomones, such as the pyridine analogs, present in the biological fluids of the predator increases the overall fitness of the species and could thus be considered as an evolutionary strategy for survival (Apfelbach et al., 2005).

## Author contributions

JB, FS, and MCB designed research; JB, FM, MNT, and MCB performed research; JB and FM analyzed data; and JB and MCB wrote the paper.

### Conflict of interest statement

The authors declare that the research was conducted in the absence of any commercial or financial relationships that could be construed as a potential conflict of interest.
